# Whole-exome analysis in osteosarcoma to identify a personalized therapy

**DOI:** 10.18632/oncotarget.19010

**Published:** 2017-07-05

**Authors:** Caterina Chiappetta, Massimiliano Mancini, Francesca Lessi, Paolo Aretini, Veronica De Gregorio, Chiara Puggioni, Raffaella Carletti, Vincenzo Petrozza, Prospero Civita, Sara Franceschi, Antonio G. Naccarato, Carlo Della Rocca, Chiara M. Mazzanti, Claudio Di Cristofano

**Affiliations:** ^1^ UOC of Pathology, Department of Medical-Surgical Sciences and Bio-Technologies, Sapienza University of Rome, Latina, Italy; ^2^ Pisa Science Foundation, Pisa, Italy; ^3^ Department of Pathology, University of Pisa, Pisa, Italy

**Keywords:** osteosarcoma, next generation sequencing, carcinogenesis, metastasis, drug resistance

## Abstract

Osteosarcoma is the most common pediatric primary non-hematopoietic bone tumor. Survival of these young patients is related to the response to chemotherapy and development of metastases. Despite many advances in cancer research, chemotherapy regimens for osteosarcoma are still based on non-selective cytotoxic drugs. It is essential to investigate new specific molecular therapies for osteosarcoma to increase the survival rate of these patients. We performed exomic sequence analyses of 8 diagnostic biopsies of patients with conventional high grade osteosarcoma to advance our understanding of their genetic underpinnings and to correlate the genetic alteration with the clinical and pathological features of each patient to identify a personalized therapy.

We identified 18,275 somatic variations in 8,247 genes and we found three mutated genes in 7/8 (87%) samples (KIF1B, NEB and KMT2C). KMT2C showed the highest number of variations; it is an important component of a histone H3 lysine 4 methyltransferase complex and it is one of the histone modifiers previously implicated in carcinogenesis, never studied in osteosarcoma. Moreover, we found a group of 15 genes that showed variations only in patients that did not respond to therapy and developed metastasis and some of these genes are involved in carcinogenesis and tumor progression in other tumors.

These data could offer the opportunity to get a key molecular target to identify possible new strategies for early diagnosis and new therapeutic approaches for osteosarcoma and to provide a tailored treatment for each patient based on their genetic profile.

## INTRODUCTION

Osteosarcoma is the most common non-haematological primary malignant tumor of the bone, it arises from mesenchymal cells that produce osteoid and immature bone and affects mainly the extremities of adolescents and young adults [1–2].

The 5-years survival rate for patients with osteosarcoma without evidence of metastasis is 60% to 65%, whereas it is only 20% to 28% for osteosarcoma patients with metastases at the time of diagnosis [3]. Treatment of high grade osteosarcoma is based on a multidisciplinary approach that includes neoadjuvant chemotherapy, surgical excision of the primary tumor and metastasis excision; evaluation of response to therapy in the surgical specimen is crucial to eventually schedule a postoperative chemotherapy [4]. Standard therapy regimens often involve the use of high-dose methotrexate, doxorubicin, cisplatin and ifosfamide [4–5]. Evaluation of response to therapy was assessed by percentage area of necrosis in the specimen after surgery; patients who respond to therapy showing tumor necrosis ≥ 90% after surgery and they do not develop metastasis (Responder) while patients that do not respond to neoadjuvant chemotherapy showing tumor necrosis ≤ 90% after surgery and eventually developing metastasis (Non-responder) [6].

Although the survival rate has improved considerably after the introduction of neoadjuvant chemotherapy and surgery, metastatic or recurrent disease still occurs and the survival rate of patients is mainly linked to the resistance to therapy and to the development of metastasis [7]. In patients with metastatic osteosarcoma treated with neoadjuvant therapy, the “Responder” status shows improved survival (82% at 5-years) compared to “Non-Responder” (70% at 5-years) [8–9]. Over the last decade, scientific research has provided essential information about the pathogenic, molecular and biochemical pathway involved in osteosarcoma [10–11]; however, molecular mechanisms related to carcinogenesis, progression and resistance to therapy are still largely unknown. Indeed, even now the osteosarcoma karyotype is considered complex and only mutations of tumor suppressors genes *TP53* and *RB1* are commonly associated with the development of osteosarcoma [12].

There are many examples of “molecular targeted therapy”, where tailored therapeutic agents have been selected to aim against specific molecules and their downstream effector pathways in each patient [13]. Nonetheless survival rates of patients have not greatly improved [14], as little is known at genetic level about the pathogenesis of osteosarcomas resulting in a lack of more effective and tailored chemotherapy drug regimens [15].

Our hypothesis is that only the analysis of the entire coding genome could lead to a better understanding of the molecular mechanisms underlying the development and progression of osteosarcoma. Next generation sequencing (NGS) technologies can unveil DNA sequences whose study can be applied to characterize both common and rare genomic alterations across cancer types. The NGS approach could clarify the landscape of genetic alteration in osteosarcoma and provide relevant biological data. In particular, the knowledge of genetic alterations in coding exon regions, through WES (Whole Exome Sequencing) approach, may result in an easier discovery of new proteins as molecular target which could be aimed at personalized therapies. Indeed, WES could lead to create algorithms to identify relevant genetic alterations networks relevant to the clinical context of the disease [16–18]. The aim of this study was to perform WES analysis in selected tumors samples of osteosarcoma, both from responder and non-responder patients that developed metastasis. The goal will be to improve the knowledge of osteosarcoma carcinogenesis, progression and resistance to therapy, to identify new strategies for therapeutic approaches and personalized therapy.

## RESULTS

### Sequence coverage and mutation analysis

This analysis provided a vast new reservoir of data and, after filtering the data, within our discovery set of eight osteosarcomas, 5 responder and 3 non-responder, we identified 18,275 somatic variations in 8,247 genes. The number of coding variations in each sample ranged from 496 to 8,500 with an average of 2,347.5 variations. All the variations were missense and the 98.5% were SNVs (Single Nucleotide Variants) while the 1.5% were MNPs (Multiple Nucleotide Polymorphisms).

**Figure 1:**
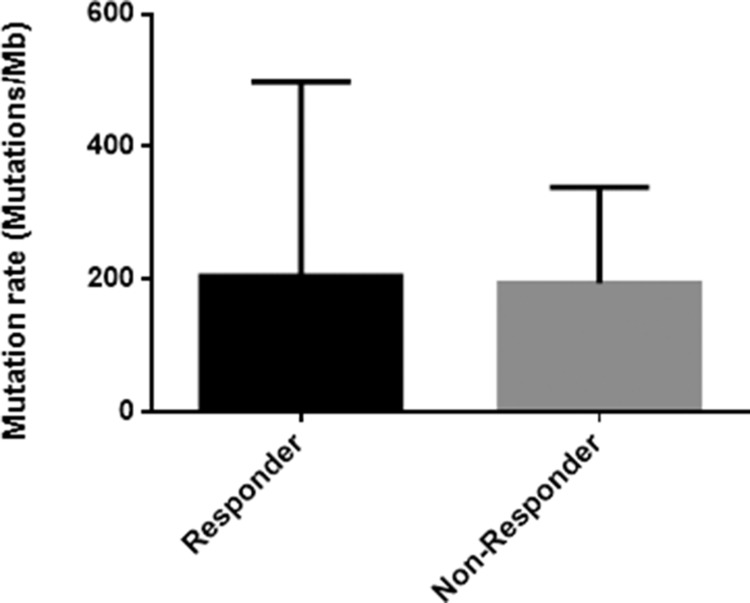
Histogram of the mutation rate in responder and non-responder group

The mutation rate in the responder group was slightly higher than the non-responder group (Table 1, Figure 1). Moreover, we found that, as in several other cancers [19], C:G>T:A transitions were the most predominant somatic substitution both in the responder group (43%) and in the non-responder group (34%) and we found that the vast majority of these transitions were identified in the context of 5′-CpG-3′ dinucleotide as the result of a 5′-methylcytosine deamination (data not shown).

**Table 1:** N° of variations and variation rate (n° of variations/Mb) of responder and non-responder osteosarcoma patients

Sample	N° of variations	Variations rate	Status
1	1390	118.8	Responder
5	8500	726.5	Responder
6	496	42.4	Responder
7	791	67.6	Responder
8	802	68.5	Responder
2	4211	359.9	Non-Responder
3	1495	127.8	Non-Responder
4	1095	93.6	Non-Responder

We did not find common mutated genes in all eight samples but we found three mutated genes (*KIF1B*, *NEB*, *KMT2C*) in 7/8 (87%) samples. Seventeen genes were mutated in 6 samples (75%, 6/8) and 57 genes showed variations in 5 samples (62.5%, 5/8). We noted a great variability of genes mutated among the samples as shown in Supplementary Table 1. Indeed, taking into consideration common mutated genes in at least 5 samples, we noted that sample n°5 showed variations in 77 genes while the sample n°6 showed variations only in 22 genes (Supplementary Table 1).

These 77 genes that were found mutated in more than half of the samples are involved in important biological processes such as transcription regulation, ion transport, DNA damage and repair, cell adhesion and angiogenesis (Table 2).

**Table 2:** Genes mutated in more than half of the osteosarcoma patients and their biological functions

Biological function	Mutated genes
Transcription regulation	ATF2; BPTF; CHD7; DENND4A; HIVEP2; IKBKAP; JMJD1C; KMT2C; KMT2E; MYCBP2; NCOR1; RFX7; SPEN;
Transport	ABCB5; ABCD2; ANKH; ATP10D; CACNA2D3; DGKD; HERC1; ITPR1; SLC8A1; SLC9B1; TRPM3;
Cell Adhesion	DST; FN1; ITGA6; MPDZ; NRXN2; RELN;
Immunity	C5; CFH; PKHD1L1;
Apoptosis	BIRC6; ITPR1; KIF1B;
Endocytosis	DGKD; LRP2; STAB2;
Cell cycle	ALMS1; ATM; BIRC6; KMT2E;
DNA damage	ATF2; ATM; DNA2; POLQ; PRDM10; PSME4;
Angiogenesis	FN1; THSD7A;
Ubl conjugation pathway	BIRC6; HECTD1; HERC1; MYCBP2;
Wnt signaling pathway	MACF1;
Differentiation	ABCB5; OBSCN; PSME4; THSD7A;
DNA repair	DNA2; POLQ; PRDM10; PSME4;
Muscle development	DMD; NEB; SYNE1; UTRN;
Regulation of bone mineralization	ENPP1; FBN2;
Cell communication	FRAS1;
mRNA processing	FRG1;
Nucleotide metabolism	NT5C2;
Multicellular organism development	PRTG;
Regulation of cell migration	SYNE2;
Small GTPase mediated signal trasduction	DOCK5;
Regulation of GTPase activity	AKAP13; DENND4A; PREX2; SYDE2;
Extracellular matrix organization	EGFLAM;
Microtuble-based movement	DNAH9; DNAH10;

Despite variations of *TP53* and *RB1* are associated with development of human osteosarcoma, we found that variations of *TP53* were present in only one patient (12.5%, sample n°5) while only two patients showed variations of *RB1* gene (25%, sample n°1 and 5).

### Genes that may be involved in osteosarcoma tumorigenesis

We found that *KIF1B*, *NEB* and *KMT2C* were the only three genes mutated in 7/8 samples. *KIF1B* is located on chromosome 1p36, and belongs to the kinesin superfamily of intermediate filaments [20]. *NEB*, maps to chromosome 2q22, encodes nebulin, a giant protein component of the cytoskeletal matrix [21]. We found 10 and 27 variations respectively in these two genes and they were mutated in all samples except in sample n°7, while sample n°5 had the higher number of variations in both cases (Table 3). Finally, *KMT2C* is located on chromosome 7q36.1 and it is a member of the mixed-lineage leukemia (MLL) family and it has histone H3 lysine-specific methylation activity. KMT2C is an important component of histone H3 lysine 4 methyltransferase complex, implicated in chromatin remodelling, called ASCOM (ASC-2-and Mll3-containing complex) [22].

**Table 3:** Somatic variation of KIF1B and NEB in osteosarcoma samples and their genomic and amino acid change

Sample	Gene	Genomic change	Aminoacid	
1	KIF1B	c.650C>T	p.Ser217Phe	
1	KIF1B	c.5074G>A	p.Asp1692Asn	
1	NEB	c.21268C>T	p.Pro7090Ser	
1	NEB	c.21197T>A	p.Leu7066His	
2	KIF1B	c.177T>A	p.His59Gln	
2	NEB	c.23803A>G	p.Arg7935Gly	
2	NEB	c.20167C>G	p.Arg6723Gly	
2	NEB	c.20167C>T	p.Arg6723Trp	
2	NEB	c.20165A>G	p.Tyr6722Cys	
2	NEB	c.20165A>T	p.Tyr6722Phe	
2	NEB	c.19249_19250delTAinsCA	p.Tyr6417His	
2	NEB	c.19063T>A	p.Tyr6355Asn	
3	KIF1B	c.2798A>G	p.Tyr933Cys	
3	NEB	c.7325T>A	p.Ile2442Asn	
4	KIF1B	c.5069A>G	p.Asp1690Gly	
4	NEB	c.11109G>C	p.Lys3703Asn	
5	KIF1B	c.538G>A	p.Asp180Asn	
5	KIF1B	c.2332G>A	p.Val778Met	
5	KIF1B	c.2344G>A	p.Glu782Lys	
5	NEB	c.23570A>G	p.Glu7857Gly	
5	NEB	c.22337T>C	p.Ile7446Thr	
5	NEB	c.22333G>C	p.Asp7445His	
5	NEB	c.22021G>C	p.Ala7341Pro	
5	NEB	c.21991G>T	p.Ala7331Ser	
5	NEB	c.21569C>T	p.Thr7190Ile	
5	NEB	c.18996G>C	p.Gln6332His	
5	NEB	c.18448G>C	p.Asp6150His	
5	NEB	c.11026G>A	p.Asp3676Asn	
5	NEB	c.8156A>T	p.Asn2719Ile	
5	NEB	c.8142T>G	p.Asn2714Lys	
5	NEB	c.2633G>C	p.Ser878Thr	
6	KIF1B	c.956C>T	p.Thr319Ile	
6	NEB	c.9705C>A	p.Asn3235Lys	
6	NEB	c.7025G>A	p.Ser2342Asn	
8	KIF1B	c.3814C>T	p.Arg1272Cys	
8	NEB	c.10286C>T	p.Thr3429Ile	
8	NEB	c.9692T>G	p.Leu3231Trp	L

Among these three genes, *KMT2C* showed the highest number of variations: we found 36 variations, with sample n°5 revealing the highest number of variations, except in sample n°3 (Table 4). Particularly, sample n°3 was a 14-year old boy with diagnosis of high grade chondroblastic osteosarcoma made on femur biopsy (stage IVa), follow-up was 11 years, he did not respond to neoadjuvant chemotherapy and develop metastasis.

**Table 4:** Somatic variations of KMT2C in osteosarcoma samples and their genomic and amino acid change

Sample	Genomic change	Aminoacid
1	c.2963G>T	p.Cys988Phe
1	c.8326G>A	p.Glu2776Lys
1	c.11927C>A	p.Pro3976Gln
1	c.12014C>T	p.Ser4005Phe
1	c.14521G>A	p.Gly4841Arg
2	c.1277C>A	p.Pro426Gln
2	c.6632G>C	p.Arg2211Thr
2	c.5459C>G	p.Ser1820Cys
2	c.8174A>G	p.Glu2725Gly
2	c.9622T>C	p.Ser3208Pro
2	c.9617G>C	p.Arg3206Thr
4	c.10383T>G	p.Asp3461Glu
5	c.404C>T	p.Ala135Val
5	c.3155A>T	p.Lys1052Ile
5	c.3119C>A	p.Pro1040Gln
5	c.3029G>T	p.Cys1010Phe
5	c.5669G>C	p.Arg1890Pro
5	c.7318C>T	p.Pro2440Ser
5	c.7214A>G	p.Glu2405Gly
5	c.9182A>G	p.Gln3061Arg
5	c.9769G>A	p.Glu3257Lys
6	c.925C>T	p.Pro309Ser
6	c.1181G>A	p.Cys394Tyr
6	c.2512G>A	p.Gly838Ser
6	c.2468T>C	p.Ile823Thr
6	c.2459C>T	p.Thr820Ile
6	c.2656C>T	p.Arg886Cys
7	c.943G>A	p.Gly315Ser
7	c.925C>T	p.Pro309Ser
7	c.2512G>A	p.Gly838Ser
7	c.2468T>C	p.Ile823Thr
7	c.2963G>T	p.Cys988Phe
7	c.2917A>G	p.Arg973Gly
8	c.943G>A	p.Gly315Ser
8	c.2512G>A	p.Gly838Ser
8	c.2468T>C	p.Ile823Thr

### Genes that may be involved in osteosarcoma metastatic progression and resistance to therapy

We found that 0.18% (15/8247) of genes showed variations, all missense (94% SNV, 6% MNP), only in the non-responder group (Table 5). Categorizing them by KEGG pathway analysis and literature review, we found that some of these are involved in important biological processes (Table 6). *ERBB4* is located on chromosome 2q34 and it is a receptor tyrosine kinase member of the epidermal growth factor receptor (EGFR) family [23]. ERBB4 is involved in biological process such as signal transduction, transport and catabolism. It is involved in human disease like cancer: it participates in pathways such as ERBB signalling, implicated in tumor migration and invasion, and in other pathway implicated in inhibition of tumor angiogenesis [24]. In signal transduction, transport and catabolism and human disease biological process is involved THBS1 too and moreover is implicated in cell growth and death process. *THBS1*, thrombospondin 1, is located on chromosome 15q14 [25] and it is involved in RAP1 and TP53 signalling pathways, involved in inhibition of angiogenesis and metastasis, in PI3K-AKT and TGF-beta signalling pathway, involved in the control of cell cycle and apoptosis, and it takes part in focal adhesion and ECM-receptor interaction process [26]. Other genes are enzymes with specific activity like as rRNA and tRNA biogenesis (*DIS3*, *KARS*), spliceosome activity (*BCLAF1*) or charboidrate metabolism (*PDHX*) and all involved in carcinogenesis [27–32].

**Table 5:** Variations of genes mutated only in non-responder group and their genomic and amino acid change

Gene	Genomic change	Aminoacid
ALDH1L2	c.484G>A	p.Gly162Ser
ALDH1L2	c.1732A>C	p.Thr578Pro
ALDH1L2	c.2086G>A	p.Gly696Ser
BCLAF1	c.491A>C	p.Lys164Thr
BCLAF1	c.1646T>C	p.Leu549Pro
BCLAF1	c.1742A>T	p.Lys581Met
CLCN1	c.823_825delGTCinsGGG	p.Val275Gly
CLCN1	c.1289A>C	p.Asn430Thr
CLCN1	c.1574C>G	p.Ala525Gly
CLCN1	c.1073G>A	p.Cys358Tyr
CLCN1	c.1427C>G	p.Thr476Ser
COG3	c.943G>C	p.Glu315Gln
COG3	c.505A>G	p.Thr169Ala
COG3	c.1411T>A	p.Tyr471Asn
DIS3	c.2312_2313delTAinsAA	p.Ile771Lys
DIS3	c.2252C>G	p.Ala751Gly
DIS3	c.2234C>T	p.Thr745Ile
DIS3	c.2458C>T	p.Arg820Trp
ERBB4	c.2761T>A	p.Tyr921Asn
ERBB4	c.1541G>A	p.Gly514Glu
ERBB4	c.1841G>A	p.Cys614Tyr
KARS	c.398T>C	p.Leu133Pro
KARS	c.1009G>T	p.Val337Phe
KARS	c.1066G>A	p.Asp356Asn
OR52N1	c.589A>G	p.Asn197Asp
OR52N1	c.569C>G	p.Ser190Cys
OR52N1	c.53G>C	p.Gly18Ala
OR52N1	c.82T>C	p.Trp28Arg
PDE6C	c.646T>A	p.Tyr216Asn
PDE6C	c.650T>C	p.Leu217Pro
PDE6C	c.1289G>A	p.Gly430Glu
PDE6C	c.1992G>T	p.Leu664Phe
PDHX	c.566G>C	p.Arg189Pro
PDHX	c.626G>A	p.Gly209Glu
PDHX	c.323G>A	p.Gly108Glu
SCN8A	c.2276T>C	p.Ile759Thr
SCN8A	c.4048G>A	p.Ala1350Thr
SCN8A	c.677G>A	p.Arg226Lys
SP140L	c.1425A>C	p.Lys475Asn
SP140L	c.329T>A	p.Leu110His
SP140L	c.1013C>A	p.Pro338His
THBS1	c.634C>G	p.Leu212Val
THBS1	c.1699_1700delGAinsGT	p.Asp567Val
THBS1	c.2365G>A	p.Asp789Asn
THBS1	c.1532G>C	p.Gly511Ala
UBE4A	c.1130T>A	p.Ile377Asn
UBE4A	c.2209G>A	p.Glu737Lys
UBE4A	c.3136G>T	p.Asp1046Tyr
ZNF12	c.137C>G	p.Ser46Cys
ZNF12	c.1265C>T	p.Ser422Phe
ZNF12	c.2086C>T	p.Leu696Phe

**Table 6:** Mutated genes in non-responder group of osteosarcoma patients and their chromosomal location and biological process

Gene	Chromosomal location	Biological process
ALDH1L2	12q23.3	Metabolism of cofactors and vitamins
BCLAF1	6q23.3	Spliceosome
CLCN1	7q34	Ion channels
COG3	13q14.13	Membrane trafficking
DIS3	13q22.1	mRNA biogenesis
ERBB4	2q34	Signal trasduction, Transport and catabolism, Human disease
KARS	16q23.1	tRNA biogenesis
OR52N1	11p15.4	Olfactory transduction
PDE6C	10q24	Sensory transduction
PDHX	11p13	Carbohydrate metabolism
SCN8A	12q13	Ion channels
SP140L	2q37.1	unknown
THBS1	15q15	Signal trasduction, Transport and catabolism, Human disease, Cell growth and death
UBE4A	11q23.3	Ubiquitin mediated proteolysis
ZNF12	7p22.1	Transcription factors

## DISCUSSION

Osteosarcoma is a heterogeneous tumor and it is a so-called “orphan cancer” with no known driver oncogenes [33]. It is essential to find useful biomarkers and to detect the potential targets for new drugs, to increase overall survival of these patients. This could be done only if we better understand the complex biology of this tumor and the molecular pathways that lead to the development of metastases and resistance to therapy. At present time, the most innovative approach is represented by NGS, which allows to analyse the entire human genome [18]. In this study, we performed the analysis of exomic sequences of osteosarcomas to advance our understanding of their genetic underpinnings and to correlate the genetic alterations with the clinical and pathological features of each patient. Our aim was to identify clinically relevant variations in the landscape of somatic events in osteosarcoma pathogenesis and so we performed WES analysis on eight high grade osteosarcoma biopsies. Using stringent criteria for the analysis of these presumptive variations, we identified 18,275 somatic variations, all missense, in 8,247 genes. We noted that the mutation rate was similar between responder and non-responder group but overall this rate was higher than previous studies probably due to the small number of our samples [34–36]. We did not find specific structural abnormalities common to all samples, such as specific translocations like other sarcomas (e.g. Ewing sarcoma EWS/FLI1 t(11;22)(q24;q12)), confirming the complexity of osteosarcoma karyotype [15]. Despite *TP53* and *RB1* genes are considered top driver genes in osteosarcoma cancerogenesis, our study showed lower mutation frequency of *TP53* and *RB1* than other studies [34,35,37–39]. Indeed, we observed that only one patients (12.5%, 1/8) showed variations of *TP53* gene and that only two patients showed variations of *RB1* gene (25%, 2/8). Our results could be the effect of the small number of samples, however the range of mutation rate described in osteosarcoma is very large going from 31% to 82% and from 19% to 64% for *TP53* and *RB1* respectively [41]. Moreover, most *TP53* mutations described in some studies, are structural variations in intron 1 [34, 37, 38] that we did not study and the mutation rate outside the intron 1 is low (19–20%) [34, 40]. However, as reported in literature, among all mutations of *TP53* reported in previous studies only some mutations are clonal events [37]. Lastly, our data was filtered by Sift and Polyphen-2 bioinformatics software, so we reported only mutations that are considered deleterious for the protein function.

However, beyond *TP53* and *RB1*, between genes considered candidate driver genes in osteosarcoma carcinogenesis [36] we found that *ATM*, a gene involved in DNA damage control signalling pathway, showed variations in 5/8 osteosarcoma samples. *ATM* is a tumor suppressor gene that belongs to the family of proteins that respond to DNA damage by phosphorylating key substrates involved in DNA repair and/or cell cycle control [36]. Moreover, we did not find variations in genes associated at inherited cancer predisposition syndromes associated with osteosarcoma as *RECQL4*, *BLM*, and *WRN* [34, 35, 37].

Moreover, we did not find variations common to all osteosarcoma samples but we found three mutated genes in 7/8 samples (87%) never studied in osteosarcoma. Variations in these three genes may be early molecular events and so they may be related to osteosarcoma tumorigenesis. KIF1B is responsible for intracellular vesicular transport [20], a process involved in the production matrix in osteosarcoma [42]. KIF1B has been shown to act as a tumor suppressor in multiple cancers by acting on various inhibitors of cell proliferation and activators of apoptosis [20]. Another interesting *NEB*, maps to chromosome 2q22, encodes nebulin, a giant protein component of the cytoskeletal matrix [21], that coexists with thick and thin filaments within the sarcomeres of skeletal muscle [21].

Finally, KMT2C catalyses the monomethylation of H3K4 in collaboration with hormone receptors and transcription factors involved in developmental signalling [22, 43]; it is one of the histone modifiers previously implicated in some cancer types [22]. It caught our attention showing more variations than *KIF1B* and *NEB*, finally because this gene is found to be inactivated in other tumors causing an alteration of tumor suppressor gene *TP53* [43], which is also frequently altered in osteosarcoma [44]. Moreover, recent evidence showed that mutations of *KMT2C* can modify its cooperation with oestrogen receptor [45], this hormone playing a key role in the development and support of bone remodelling and matrix production [46]. Recently, it has been demonstrated that highly conserved epigenetic regulators are frequently mutated in cancer [47]; indeed, some studies suggest that variations in the coding sequences of regulatory elements, that act on enhancers to recognize specific transcription factors, could be the cause of tumor development [47]. KMT2C acts in collaboration with hormone receptors and transcription factors involved in growth-promoting pathways and it performs its action at transcription enhancer regions [22]. *KMT2C* is frequently mutated in a broad spectrum of cancers and it has been related to tumorigenesis [48]. Therefore, our hypothesis is that KMT2C could be involved in osteosarcoma carcinogenesis too.

We found that the responder group showed more variations than the non-responder group and we noted that 15 genes showed variations only in the non-responder group which subsequently developed a metastatic disease. We found that some of them are involved in carcinogenesis and tumor progression but not in osteosarcoma. Particularly, ERBB4 is a member of the Tyr protein kinase family and the epidermal growth factor receptor subfamily that regulates cell proliferation and differentiation [23]. It has been shown that ERBB4 is overexpressed in Ewing Sarcoma cell lines derived from chemoresistant or metastatic Ewing sarcoma, a tumor of the bone [24]. ERBB4 leads to activation of PI3K-AKT, focal adhesion kinase (FAK) and the RAC1 GTPase, a mediator of cell migration and invasion [24], pathways that are already been described in osteosarcoma pathogenesis [49–50].

*ERBB4* is rarely mutated in human cancers [51] but recently it was shown that ERBB4 can inhibit the tumor suppressor TP53 by regulating the MDMX-MDM2 complex stability, the primary inhibitors of TP53, leading to TP53 inactivation in tumor [52]. Thrombospondin I (THBS1) is an endogenous inhibitor of angiogenesis, which limits blood vessel density in normal tissues and curtails tumor growth. It interacts with a variety of ECM molecules and cell surface receptors and this protein has been shown to play roles in tumorigenesis; particularly, it was showed that THBS1 induced cell migration in several tumor cell lines suggesting that THBS1 is involved in cancer invasion [26]. DIS3 is one of catalytic components of the human RNA processing and degrading exosome it has both endonucleolytic and 3-prime/5-prime exonucleolytic activity [27]; mutations in *DIS3* have been observed in several cancer types [28] and in multiple myeloma was observed a shorter survival in patients with *DIS3* mutations [29]. *BCLAF1* encodes a transcriptional repressor that interacts with several members of the BCL2 family of proteins and ectopical *BCLAF1* expression induces apoptosis in various cell types or autophagic cell death in myeloma cells. It was hypothesized that BCLAF1 plays a critical role repressing the transcription of survival genes through TP53 inhibition. Moreover, it was found that tumor cells suppress *BCLAF1* expression inducing a cascade of antiapoptotic cellular events that all together determine an increase survival of tumor cells [31]. The *KARS* gene encodes lysyl-tRNA synthetase, which catalyses the aminoacylation of tRNA-lys in the cytoplasm and mitochondria. It was showed that it was implicated in cancer metastasis indeed the phosphorylation of KARS at the Thr52 residue by p38MAPK, causes the dissociation from the cytosolic multi-tRNA synthetase complex and the following translocation to the plasma membrane, where it associates with a 67 kDa laminin receptor (p67LR) involved in migration and metastasis [30]. The *PDHX* gene encodes component X of the pyruvate dehydrogenase (PDH) complex that is in the mitochondrial matrix and catalyzes the conversion of pyruvate to acetyl coenzyme A [32]. It was showed that in colorectal cancer cells the glucose metabolism was regulated by miR-26a direct targeting the PDHX, which inhibits the conversion of pyruvate to acetyl coenzyme A in the citric acid cycle to require the glucose uptake to the energy needs of cancer cells [32]. In successive studies, it will be necessary to study whether and how these mutations may modify the expression of these genes and the function of the associated protein to discovery a possible molecular target.

Some of these genes identified in our study are involved in metastatic progression and poor survival in tumors, also of mesenchymal origin, but these have never been studied in osteosarcoma [24, 26, 29, 30]. Our hypothesis is that alterations in these genes could be involved in a mechanism of metastatic progression and drug resistance in osteosarcoma.

We recognize these preliminary results may arise due to the small sample size, which is a limitation of the study, and we believe that it will be necessary first to ascertain our data in a larger cohort of patients.

In conclusion, our WES analysis confirms that the osteosarcoma karyotype is complex, however, we found genes probably involved in osteosarcoma carcinogenesis, as *KMT2C*, and we identified a group of 15 genes probably involved in metastasis development and drug resistance that could offer the opportunity to identify potential drug targets in order to create a personalized therapy and increase the survival rate among young patients with osteosarcoma.

## MATERIALS AND METHODS

### Population study

The population of this study included 8 diagnostic biopsies of patients with conventional high grade osteosarcoma (sec. Enneking classification) obtained before neoadjuvant therapy from the files of the Department of Medical and Surgical Sciences and Biotechnologies, Sapienza University of Rome, Polo Pontino, ICOT, Latina, Italy. The WHO 2008 classification of bone tumors was used for classifying and staging the bone lesions. Patients had a mean follow-up time of 9 years (range 1–14 years), 50% of them showed a minimum 5-years overall survival. Evaluation of response to therapy was assessed by percentage area of necrosis in the specimen after surgery; out of these 8 patients, 5 (identified as “responder”) responded to therapy showing tumor necrosis ≥ 90% after surgery and didn't develop metastasis while 3 patients did not respond to neoadjuvant chemotherapy showing tumor necrosis ≤ 90% after surgery and developed metastasis (identified as “non-responder”). There were not inherited cancer predisposition syndromes associated with osteosarcoma among our samples. The clinical and pathological characteristics of each osteosarcoma patients are described in Table 7. All the tissue samples were reviewed on microscopy examinations by two dedicated pathologists.

**Table 7:** Clinicopathological characteristics of 8 osteosacoma patients

Sample	Sex	Age	Histological type*	Grading**	Staging*	Site	Follow-up (Years)	Status
1	F	29	Conventional Osteosarcoma	High grade	IIB	Jaw	14	Responder
2	M	30	Conventional Osteosarcoma	High grade	III	Femur	3	Non-Responder
3	M	14	Chondroblastic osteosarcoma	High grade	IVA	Femur	11	Non-Responder
4	M	35	Conventional Osteosarcoma	High grade	III	Femur	1	Non-Responder
5	M	21	Conventional Osteosarcoma	High grade	III	Femur	13	Responder
6	M	11	Conventional Osteosarcoma	High grade	III	Femur	5	Responder
7	F	24	Conventional Osteosarcoma	High grade	IIB	Mandible	12	Responder
8	M	31	Chondroblastic osteosarcoma	High grade	IIA	Jaw	13	Responder

*Sec. WHO 2008

** Sec. Enneking grade

### Whole-exome sequencing (WES)

Genomic DNA was extracted from FFPE osteosarcoma biopsies using RecoverAll Total Nucleic Acid isolation FFPE kit (Life Technologies, Foster City, CA) according to the manufacturer's instruction and was used to prepare fragment libraries suitable for massively parallel paired-end sequencing.

DNA was amplified with GenomePlex Single Cell Whole Genome Amplification kit, following the manufacturer's protocol, because of the degraded material, typical of osteosarcoma samples. We measured DNA concentration of the samples using Qubit 2.0 Fluorometer (Invitrogen, Life Technologies, Grand Island, NY) with the Qubit DNA HS assay kit or Qubit DNA BR Assay kit according to the quantity of the starting material. Moreover, we checked the quality of DNA with 2200 Tapestation Instrument (Agilent Technologies, Santa Clara, CA, USA) using the D1000 screen tape or the High sensitivity D1000 screen tape as appropriate, to see the level of fragmentation of our samples.

To prepare the DNA library we used Nextera Rapid Capture Expanded Exome Kit (Illumina, San Diego, CA, USA) following the guidelines of the protocol but we used the half of the amount of the fragmentation enzyme because of the high level of fragmentation of all the samples tested with Tapestation. We load a maximum of 5 pooling libraries for each cartridge NextSeq High Output (300 cycles) on NextSeq500 (Illumina, San Diego, CA, USA).

### WES data analysis

We sequenced the whole exomes of eight high-grade osteosarcoma tissues using NextSeq550 Illumina platform. The instrument generated approximately an average read length of 120.6 bases. After mapping to the human reference genome GRCh37 (hg19) using Burrows-Wheeler Alignment tool (BWA), we obtained the average depth of each base in the target regions as 17.7x. The average coverage of target regions was 121.7x.

The raw data generated from Illumina NextSeq550 were converted using Bcl2toFastq tools provided by Illumina. The data analysis of exomes was performed by using the SeqMule pipeline1. The final Variant Call Format files (VCFs) were uploaded on the VariantStudio Illumina (Illumina San Diego, CA, USA) software to perform annotation and filtration steps. We filtered genomic data by quality score of 30, by mean read depth of 5, and by GMAF ≤ 1 (Global Minor Allele Frequency: the frequency of the second most frequent allele in the population). Next, the detected mutations were filtered for the variants absent in database of Single Nucleotide Polymorphisms (dbSNP) to distinguish known somatic mutations from germline mutations. We further selected the somatic variations in the coding sequence by excluding those variations in flanking sequences like splicing sites, 5ʹ-UTR, 3ʹUTR, introns and intergenic regions. Moreover, we performed bioinformatics analysis using SIFT (v4.0.3) and PolyPhen-2 (v2.1.0) tools, that predict whether an amino acid substitution affects protein function based on sequence homology. To be sure that the variations to study were only those that caused a protein alteration, we selected only those that were at the same time deleterious for SIFT and probably damaging for PolyPhen-2.

### Validation of variations

Genes that showed variation in the 87% of osteosarcoma samples (*KIF1B*, *NEB* and *KMT2C*) were selected for target resequencing and validation by Sanger sequencing. DNAs were amplified using the Gene Amp PCR System 9700 (Life Technologies, Foster City, CA). The PCR products were then purified using Exosap-IT (Affymetrix, Santa Clara, CA) and then sequenced using Big Dye Terminator version 1.1 Cycle Sequencing Kit (Life Technologies, Foster City, CA). Unincorporated primers and dye terminators were removed using the Montage-SEQ96 Sequencing Reaction Cleanup Kit (Merck Millipore, Billerica, MA). Sequencing was performed on an ABI PRISM 3100 Genetic Analyzer (Life Technologies, Foster City, CA) with 3100 Genetic Analyzer Data Collection software version 1.1. The sequencing and each reaction were performed in triplicate.

## SUPPLEMENTARY TABLE





## Abbreviations

NGS: Next Generation Sequencing
WES: Whole Exome Sequencing
BWA: Burrows-Wheeler Alignment
SNV: Single Nucleotide Variant
MNP: Multiple Nucleotide Polymorphisms
KIF1B: Kinesin Family member 1B
NEB: Nebulin
KMT2C: lysine (K)-specific Methyltransferase 2C
TP53: Tumor Protein 53
RB1: Retinoblastoma 1
MLL: Mixed-Lineage Leukemia
ASCOM: ASC-2-and Mll3-containing complex
KEGG: Kyoto Encyclopedia of Genes and Genomes
ERBB4: Erb-b2 receptor tyrosine kinase 4
EGFR: Epidermal Growth Factor Receptor
THBS1: Thrombospondin 1
RAP1: (Ras-proximate-1)
PI3K: Phosphoinositide 3-Kinase
AKT: Serine/Threonine Kinase 1
TGF-BETA: transforming growth factor, beta 1
ECM: Extra-Cellular Matrix
DIS3: DIS3 homolog, exosome endoribonuclease and 3′-5′ exoribonuclease
KARS: lysyl-tRNA synthetase
BCLAF1: BCL2 associated transcription factor 1
PDHX: pyruvate dehydrogenase complex component X
EWS: EWS RNA binding protein 1
FLI1: Fli-1 proto-oncogene, ETS transcription factor
FAK: Focal Adhesion Kinase
RAC1: ras-related C3 botulinum toxin substrate 1
GTP: Guanosine Triphosphate
MDMX: MDM4, p53 regulator
MDM2: MDM2 proto-oncogene
p38MAPK: p38 Mitogen-Activated Protein Kinases
p67LR: Laminin Receptor
PHD: Pyruvate Dehydrogenase
FFPE: Formalin-Fixed Paraffin Embedded
VCF: Variant Call Format
GMAF: Global Minor Allele Frequency
UTR: Untraslated Region
