# Supplementary material for: Whole-exome analysis in osteosarcoma to identify a personalized therapy
Source: Oncotarget. 2017 Jul 5;8(46):80416–28. doi: 10.18632/oncotarget.19010 (PMC5655208; doi:10.18632/oncotarget.19010)
Supplement: Supplementary file 1 [file oncotarget-08-80416-s001.pdf]

Whole-exome analysis in osteosarcoma to identify a personalized therapy

Supplementary Materials

Supplementary Table 1: Mutated genes in the 87% (7/8), 75% (6/8) and 62.5% (5/8) of osteosarcoma patients and their chromosomal location. The samples showing the mutated gene are highlighted in gray. See Supplementary_Table_1
