# Supplementary material for: Whole-exome analysis in osteosarcoma to identify a personalized therapy
Source: Oncotarget. 2017 Jul 5;8(46):80416–28. doi: 10.18632/oncotarget.19010 (PMC5655208; doi:10.18632/oncotarget.19010)
Supplement: Supplementary file 2 [file oncotarget-08-80416-s002.docx]

| **Supplementary Table 1. Mutated genes in the 87% (7/8), 75% (6/8) and 62.5% (5/8) of osteosarcoma patients and their chromosomal location. The samples showing the mutated gene are highlighted in gray** | | |  |  |  |  |  |  |  |  |
| --- | --- | --- | --- | --- | --- | --- | --- | --- | --- | --- |
|  |  |  |  |  |  |  |  |  |  |  |
| **N° of mutated samples (%)** | **Gene** | **Chromosomal location** | **Sample n°1** | **Sample n°2** | **Sample n°3** | **Sample n°4** | **Sample n°5** | **Sample n°6** | **Sample n°7** | **Sample n°8** |
| **7/8 (87%)** | **KIF1B** | **1p36.2** |  |  |  |  |  |  |  |  |
|  | **NEB** | **2q22** |  |  |  |  |  |  |  |  |
|  | **KMT2C** | **7q36.1** |  |  |  |  |  |  |  |  |
| **6/8 (75%)** | **MYCBP2** | **13q22** |  |  |  |  |  |  |  |  |
|  | **PKHD1L1** | **8q23** |  |  |  |  |  |  |  |  |
|  | **RFX7** | **15q21.3** |  |  |  |  |  |  |  |  |
|  | **SYNE1** | **6q25** |  |  |  |  |  |  |  |  |
|  | **ABCD2** | **12q12** |  |  |  |  |  |  |  |  |
|  | **C5** | **9q33-q34** |  |  |  |  |  |  |  |  |
|  | **LPHN2** | **1p31.1** |  |  |  |  |  |  |  |  |
|  | **STAB2** | **12q23.3** |  |  |  |  |  |  |  |  |
|  | **AKAP13** | **15q25.3** |  |  |  |  |  |  |  |  |
|  | **DST** | **6p12.1** |  |  |  |  |  |  |  |  |
|  | **BPTF** | **17q24.3** |  |  |  |  |  |  |  |  |
|  | **SLC8A1** | **2p22.1** |  |  |  |  |  |  |  |  |
|  | **FN1** | **2q34** |  |  |  |  |  |  |  |  |
|  | **USH2A** | **1q41** |  |  |  |  |  |  |  |  |
|  | **UTRN** | **6q24** |  |  |  |  |  |  |  |  |
|  | **VPS13D** | **1p36.22** |  |  |  |  |  |  |  |  |
|  | **CDH7** | **8q12.2** |  |  |  |  |  |  |  |  |
| **5/8 (62.5%)** | **CFH** | **1q32** |  |  |  |  |  |  |  |  |
|  | **DIP2B** | **12q13.12** |  |  |  |  |  |  |  |  |
|  | **DNA2** | **10q21.3-q22.1** |  |  |  |  |  |  |  |  |
|  | **DOCK5** | **8p21.2** |  |  |  |  |  |  |  |  |
|  | **ENPP1** | **6q22-q23** |  |  |  |  |  |  |  |  |
|  | **ITPR1** | **3p26.1** |  |  |  |  |  |  |  |  |
|  | **NBEA** | **13q13** |  |  |  |  |  |  |  |  |
|  | **PREX2** | **8q13.2** |  |  |  |  |  |  |  |  |
|  | **RELN** | **7q22** |  |  |  |  |  |  |  |  |
|  | **TRPM3** | **9q21.12** |  |  |  |  |  |  |  |  |
|  | **ANKH** | **5p15.1** |  |  |  |  |  |  |  |  |
|  | **CACNA2D3** | **3p21.1** |  |  |  |  |  |  |  |  |
|  | **FBN2** | **5q23.3** |  |  |  |  |  |  |  |  |
|  | **FRAS1** | **4q21.21** |  |  |  |  |  |  |  |  |
|  | **NT5C2** | **10q24.32** |  |  |  |  |  |  |  |  |
|  | **PSME4** | **2p16.2** |  |  |  |  |  |  |  |  |
|  | **SYNE2** | **14q23.2** |  |  |  |  |  |  |  |  |
|  | **ANK2** | **4q25-q27** |  |  |  |  |  |  |  |  |
|  | **ATF2** | **2q32** |  |  |  |  |  |  |  |  |
|  | **ATP10D** | **4p12** |  |  |  |  |  |  |  |  |
|  | **DENND4A** | **15q22.31** |  |  |  |  |  |  |  |  |
|  | **DNAH9** | **17p12** |  |  |  |  |  |  |  |  |
|  | **ATM** | **11q22-q23** |  |  |  |  |  |  |  |  |
|  | **DGKD** | **2q37.1** |  |  |  |  |  |  |  |  |
|  | **MERTK** | **2q14.1** |  |  |  |  |  |  |  |  |
|  | **NCOR1** | **17p11.2** |  |  |  |  |  |  |  |  |
|  | **NRXN2** | **11q13** |  |  |  |  |  |  |  |  |
|  | **FRG1** | **4q35** |  |  |  |  |  |  |  |  |
|  | **HYDIN** | **16q22.2** |  |  |  |  |  |  |  |  |
|  | **MUC6** | **11p15.5** |  |  |  |  |  |  |  |  |
|  | **BIRC6** | **2p22.3** |  |  |  |  |  |  |  |  |
|  | **HIVEP2** | **6q23-q24** |  |  |  |  |  |  |  |  |
|  | **OBSCN** | **1q42.13** |  |  |  |  |  |  |  |  |
|  | **EGFLAM** | **5p13.2-p13.1** |  |  |  |  |  |  |  |  |
|  | **ITGA6** | **2q31.1** |  |  |  |  |  |  |  |  |
|  | **POLQ** | **3q13.33** |  |  |  |  |  |  |  |  |
|  | **HECTD1** | **14q12** |  |  |  |  |  |  |  |  |
|  | **MACF1** | **1p32-p31** |  |  |  |  |  |  |  |  |
|  | **PRDM10** | **11q25** |  |  |  |  |  |  |  |  |
|  | **DMD** | **Xp21.2** |  |  |  |  |  |  |  |  |
|  | **LRP2** | **2q31.1** |  |  |  |  |  |  |  |  |
|  | **CEP120** | **5q23.2** |  |  |  |  |  |  |  |  |
|  | **DNAH10** | **12q24.31** |  |  |  |  |  |  |  |  |
|  | **JMJD1C** | **10q21.3** |  |  |  |  |  |  |  |  |
|  | **MDN1** | **6q15** |  |  |  |  |  |  |  |  |
|  | **MPDZ** | **9p23** |  |  |  |  |  |  |  |  |
|  | **RTTN** | **18q22.2** |  |  |  |  |  |  |  |  |
|  | **ABCB5** | **7p21.1** |  |  |  |  |  |  |  |  |
|  | **ALMS1** | **2p13** |  |  |  |  |  |  |  |  |
|  | **HERC1** | **15q22** |  |  |  |  |  |  |  |  |
|  | **IKBKAP** | **9q31** |  |  |  |  |  |  |  |  |
|  | **KMT2E** | **7q22.1** |  |  |  |  |  |  |  |  |
|  | **PRTG** | **15q21.3** |  |  |  |  |  |  |  |  |
|  | **SLC9B1** | **4q24** |  |  |  |  |  |  |  |  |
|  | **SPEN** | **1p36** |  |  |  |  |  |  |  |  |
|  | **SYDE2** | **1p22.3** |  |  |  |  |  |  |  |  |
|  | **THSD7A** | **7p21.3** |  |  |  |  |  |  |  |  |
